# Mean arterial pressure targeted fluid resuscitation may lead to fluid overload: A bleeding-resuscitation animal experiment

**DOI:** 10.1371/journal.pone.0196188

**Published:** 2018-06-28

**Authors:** Nándor Öveges, Ildikó László, Krisztián Tánczos, Márton Németh, Gábor Lebák, Bianca-Andreea Tudor-Drobjewski, Dániel Érces, József Kaszaki, László Rudas, Wolfgang Huber, Zsolt Molnár

**Affiliations:** 1 Department of Anesthesiology and Intensive Therapy, University of Szeged, Szeged, Hungary; 2 Department of Anesthesiology and General Intensive Care Medicine, Medical University of Vienna, Vienna, Austria; 3 Institute of Surgical Research, University of Szeged, Szeged, Hungary; 4 Technische Universität München, Klinikum rechts der Isar, Medizinische Klinik und Poliklinik II, München, Germany; Max Delbruck Centrum fur Molekulare Medizin Berlin Buch, GERMANY

## Abstract

**Introduction:**

Fluid resuscitation is the cornerstone of treatment in hemorrhagic shock. Despite increasing doubts, several guidelines recommend to maintain mean arterial pressure (MAP) >65 mmHg as the most frequent indication of fluid therapy. Our aim was to investigate the effects of a MAP-guided management in a bleeding-resuscitation animal experiment.

**Materials and methods:**

After anesthesia and instrumentation (t_bsl_) animals were bled till the initial stroke volume index dropped by 50% (t_0_). Fluid replacement was performed in 4 equivalent steps (t_1-4_) with balanced crystalloid solution to reach the baseline values of MAP. Invasive hemodynamic measurements and blood gas analyses were performed after each step.

**Results:**

Mean arterial pressure dropped from t_bsl_ to t_0_ (114±11 vs 76.9±16.9 mmHg, *p*<0.001) and returned to baseline by t_4_ (101.4±14.4 mmHg). From t_bsl_-t_0_ stroke volume index (SVI), cardiac index (CI) decreased (SVI: 40±8.6 vs 19.3±3.6 ml/m^2^, *p*<0.001; CI: 3.4±0.3 vs 1.9±0.3 l/min/m^2^, *p*<0.001), pulse pressure variation (PPV) increased (13.2±4.3 vs 22.1±4.3%, *p*<0.001). There was a decrease in oxygen delivery (464±45 vs 246±26.9 ml/min, *p*<0.001), central venous oxygen saturation (82.8±5.4 vs 53.6±12.1%, *p*<0.001) and increase in lactate levels (1.6±0.4 vs 3.5±1.6 mmol/l, *p*<0.005). SVI, CI and PPV returned to their initial values by t_2_. To normalize MAP fluid therapy had to be continued till t_4_, with the total infused volume of 4.5±0.8 l.

**Conclusion:**

In the current experiment bleeding led to hemorrhagic shock, while MAP remained higher than 65 mmHg. Furthermore, MAP was unable to indicate the normalization of SVI, CI and PPV that resulted in unnecessary fluid administration. Our data give further evidence that MAP may be an inappropriate parameter to follow during fluid resuscitation.

## Introduction

Acute blood loss results in an imbalance between oxygen delivery (DO_2_) and consumption (VO_2_), which can later lead to shock and severe organ dysfunction [1,2]. Cumulative oxygen debt has also been shown to have a direct relationship with hospital mortality [3]. Therefore, from a clinician’s perspective, the three main principles of managing any type of circulatory shock are: identifying the type of shock, selecting the appropriate therapy and evaluating the patient’s response to the therapy [4].

During the early phase of treatment of hemorrhagic shock fluid replacement plays a pivotal role. However, fluid resuscitation is a double-edged sword. Administering too little can cause hypoperfusion, while excessive fluid loading can lead to tissue edema, both causing organ failure, hence increased morbidity and mortality [5, 6]. Therefore, keeping the patients normovolemic should be mandatory in order to avoid harm, but it remains uncertain, which parameter(s) should we follow to guide fluid therapy during resuscitation.

From the physiological perspective the rationale of fluid resuscitation during bleeding/hypovolemia is to increase stroke volume, hence cardiac output, and eventually DO_2_. Traditionally mean arterial pressure (MAP) and central venous pressure (CVP) have been used most frequently to guide fluid management. However, there are several publications showing that neither MAP nor CVP are reliable predictors of fluid responsiveness, hence for guiding fluid therapy [7, 8, 9]. Despite this large body of evidence, MAP and CVP has still been recommended to be used for fluid management at the bedside by several guidelines [10, 11]. Indeed, a recent survey showed that almost 60% of intensive care physicians still consider hypotension (i.e.: MAP) as their primary indicator for assessing fluid responsiveness and indication for fluid resuscitation [12]. Despite the routine application of MAP at the bedside, the effects of MAP-guided fluid resuscitation on the hemodynamic profile (i.e.: CO and VO_2_/DO_2_) have not been investigated in detail yet.

Therefore, the aim of the current experiment was to investigate the effects of a MAP-normalization-targeted resuscitation on hemodynamic indices and VO_2_/DO_2_ in hemorrhagic shock in an animal model.

## Materials and methods

The experiments were carried out in strict adherence to the National Institute of Health guidelines for the use of experimental animals, and the study was approved by the National Scientific Ethical Committee on Animal Experimentation (National Competent Authority), with the license number: V./142/2013.

### Animals

The experimental subjects were 10 Vietnamese pot-bellied pigs of both sexes (1 male, 9 females) weighing 33±4 kg, provided by a certified local breeder. Each animal has a 1.4m^2^ area equipped with individual feeder and watering system. For small herds including 2–3 pigs, 2 paddocks are available, both with an area of 3.8 m^2^. Standard animal fodder is used for animal nutrition. Satisfactory ventilation, air-conditioning and heating is provided. Cardboard (couching), halved tree trunk (scratching), small, soft tree pieces and corn cob (rummaging) are used as environmental enrichment. The animal caretakers have 8–10 years of experience. The supervisor of the animal accommodation holds a degree in general medicine. Veterinarian aid is provided in case of need.

### Instrumentation

The animals underwent a 12 hours fast pre-operatively but had free access to water. Anesthesia was induced by intramuscular injection of a mixture of ketamine (20 mg/kg) and xylazine (2 mg/kg) and maintained with a continuous iv. infusion of Propofol (50 μL/min/kg IV; 6 mg/kg/hr), while analgesia was maintained with intermittent nalbuphine (0.1 mg/kg). After endotracheal intubation the animals were mechanically ventilated with a Hamilton C1 respirator (Hamilton Medical AG, USA). The tidal volume was set to 10 ml/kg, and the respiratory rate was adjusted to maintain the end-tidal carbon dioxide and partial pressure of arterial carbon dioxide within the range of 35–45 mmHg and the arterial pH between 7.35 and 7.45. The depth of anesthesia was assessed by checking jaw tone. After induction of anesthesia, catheters were inserted into the left jugular vein, left external carotid artery and the left femoral artery. For invasive hemodynamic monitoring, a transpulmonary thermodilution catheter (PiCCO, PULSION Medical Systems SE, Munich, Germany) was used. The femoral artery served as the site for arterial blood gas sampling, the central venous line was used for taking central venous blood gas samples and for the injection of cold saline boluses for the thermodilution measurements, whilst the carotid arterial catheter was used for draining blood. A transpubic catheter was placed into the urinary bladder for monitoring renal function. Animals were covered in scrubs and an external heating device was used to maintain physiological body temperature.

### Hemodynamic and blood gas measurements

Stroke volume (SV), heart rate (HR), mean arterial pressure (MAP), central venous pressure (CVP), cardiac output (CO), global end-diastolic volume (GEDV), SV variation (SVV), pulse pressure variation (PPV), left ventricular contractility (dPmax) and systemic vascular resistance (SVR) were measured by transpulmonary thermodilution and/or pulse contour analysis at baseline and after every achieved target value. All hemodynamic parameters were indexed to body surface area. The average of two measurements following 10-ml bolus injections of ice-cold normal saline were recorded.

From the arterial and central venous blood gas samples (Cobas b 221, Roche Ltd, Basel, Switzerland) that were drawn and analyzed by co-oximetry simultaneously at baseline and at the end of each step, ScvO_2_ and dCO_2_ were determined. From these parameters, the following variables were calculated:

$$DO_{2}=CI\times (Hb\times 1.34\times SaO_{2}+0.003\times PaO_{2})$$

$$DO_{2}=CI\times CaO_{2}$$

$$VO_{2}=CI\times \left(CaO_{2}-\left(Hb\times 1.34\times ScvO_{2}+0.003\times PcvO_{2}\right)\right)Oxygen\;Extraction=VO_{2}÷DO_{2}$$

### Experimental protocol

**Fig 1:**
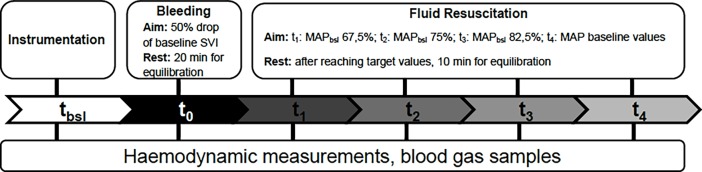
Experimental protocol.

The experimental protocol is delineated in Fig 1. After instrumentation, animals were allowed to rest for 30 minutes, after which baseline (t_bsl_) hemodynamic measurements, blood gas analyses and laboratory testing were performed. After baseline data were recorded, blood was drained from the arterial catheter placed in the left external carotid artery by direct suctioning with a 50 mL syringe, until the SV index (SVI) dropped by 50% of its initial value (t_0_), then measurements were repeated. After reaching the target reduction of SVI, values of MAP were taken as targets of fluid replacement during the rest of the study. The difference between MAP_tbsl_−MAP_t0_ was divided equally into four target values, which were aimed to be achieved in four steps during fluid resuscitation (t_1–4_) to reach the baseline MAP by t_4_. Fluid loading was carried out with boluses of balanced crystalloid Ringer Fundin (B. Braun AG., Melsungen, Germany). For practical reasons the total amount of crystalloid to be used for resuscitation was limited to 4.5 times the volume of the drained blood. After reaching each step, 10 minutes were allowed for equilibrium, then hemodynamic and blood gas parameters were measured. At the end of resuscitation, the animals were euthanized with Na-pentobarbital (120 mg/kg) while still under anesthesia (dx.doi.org/10.17504/protocols.io.mrac52e)

### Data analysis and statistics

Data are presented as mean ± standard deviation unless indicated otherwise. For testing normal distribution, the Shapiro-Wilk test was used. Changes in all parameters throughout the experiment were tested by repeated measures analysis of variance. For pairwise comparisons, Pearson’s correlation was used. For statistical analysis, Statistical Program for Social Sciences version 23.0 for Windows (SPSS, Chicago, IL, USA) was used, and *p* <0.05 was considered statistically significant.

## Results

### Macro-hemodynamics

**Fig 2:**
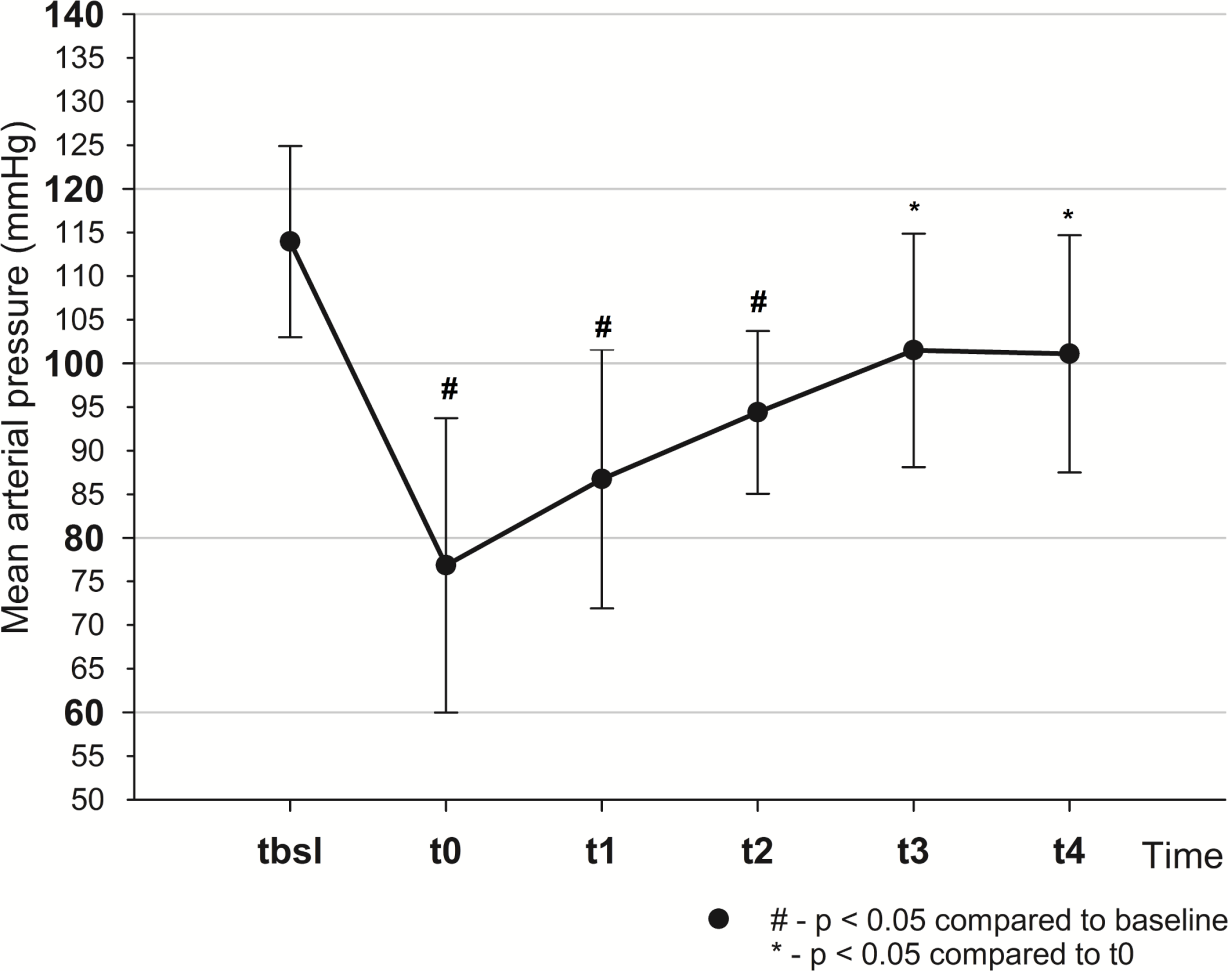
Changes of mean arterial pressure throughout the experiment.

During bleeding, a total of 712±179 ml of blood had to be drained to attain a 50% drop in SVI. For fluid replacement, a total of 3277±1223 ml of Ringer Fundin solution was administered to reach the target value of MAP obtained at t_bsl_. Hemodynamic changes during the study are summarized in Table 1. After bleeding, the SVI decreased by the planned 50% by t_0_. Mean arterial pressure dropped significantly by t_0_ and increased gradually by t_4_, although, MAP at t_4_ remained lower when compared to t_bsl_ but this difference did not reach statistical significance (Fig 2).

**Table 1:** Hemodynamic and blood gas parameters.

n = 10						
	t_bsl_	t_0_	t_1_	t_2_	t_3_	t_4_
**PPV** (%)	13.2±4.3	22.1±4.3^a^	18.3±4.7	15.1±3.3^b^	14.1±6.9^b^	12.3±3.5^b^
**GEDVI** (ml/m^2^)	349±50	282±37^a^	330±25^a^	393±56^b^	419±76^b^	440±76^b^
**CFI** (min^−1^)	9.7±1	8.4±1	10.1±2.4	11±2.4	10.2±2.4	11.4±2.8^b^
**GEF** (%)	46±8	34±4^a^	41±6	48±7^b^	46±5^b^	50±7^b^
**dPmx** (mmHg/s)	514±109	486±118	631±107	768±120^a^^b^	649±72	706±159^a^^b^^b^
**EVLWI** (ml/kg)	7.5±2	6.3±1.1	6.7±0.7	7.4±0.6	7.7±0.8	7.6±0.7
**SVV** (%)	13.3±4.3	15.4±2.9	16.3±3.8	16±2.1	14.3±7	13.9±4.9
**SVRI** (dyn×s/cm5/m^2^)	2784±451	3503±1291	2724±965	2333±793	2663±1370	2163±910
**CVP** (mmHg)	4.4±2.5	3.6±2.6	3.3±2.9	4.1±1.4	4.3±1.5	4.7±2.7
**HR** (beats/min)	84±27	94±12	94±20	87±13	86±18	90±24
**ScvO**_**2**_ (%)	83±5^b^	54±12^a^	55±10^a^	55±14^a^	61±8^a^	63±13^a^
**CO**_**2**_**g** (mmHg)	6.1±3.2	14.4±5.3^a^	12.2±3.9	9.1±4.2	8.8±3.9	8.4±3.8^b^
**aHb** (mmol/l)	10±1	9.5±1.6	8.5±1.7	7±1.4^a^^b^	7.3±1.8^a^^b^	6.6±1.2^a^^b^
**VO**_**2**_ (ml/min)	76±34	107±20	131±29^a^	146±44^a^	119±25	125±60
**DO**_**2**_ (ml/min)	464±45	246±27^a^	298±41^a^	333±38^a^^b^	308±47^a^^b^	350±45^a^^b^
**VO**_**2**_**/DO**_**2**_ (%)	16.8±7.3	46.3±11.5^a^	45.9±9.9^a^	44.6±13.3^a^	39.7±7.3^a^	35.7±13.9^a^
**Lac** (mmol/l)	1.6±0.4	3.5±1.6^a^	3.1±1.7	2.1±0.8	1.7±0.7	1.5±0.6^b^
**BE** (mmol/l)	5.3±1.5	3.7±1.8	4.1±1.5	4.5±2.1	3.5±1.9	3.7±1.2
**UO** (ml)	367±288	79±43	60±70^a^	91±88	62±91^a^	486±401^b^

Data are shown as mean±standard deviation. For statistical analyses, Shapiro-Wilk normality test and repeated measures ANOVA were used.

^a^—*p* < 0.05 compared to baseline

^b^—*p* < 0.05 compared to t_0_

PPV—pulse pressure variation; GEDVI—global end diastolic volume index; CFI—cardiac function index; GEF—global ejection fraction; dPmx—left ventricular contractility; EVLWI–extravascular lung water index; SVV–stroke volume variation; SVRI–systemic vascular resistance index; CVP–central venous pressure, HR–heart rate; ScvO_2_ –central venous oxygen saturation; CO_2_g –carbon-dioxide gap; aHb–arterial hemoglobine; VO_2_ –oxygen consumption; DO_2_ –oxygen delivery; Lac–lactate; BE–base excess; UO–urinary output.

**Fig 3:**
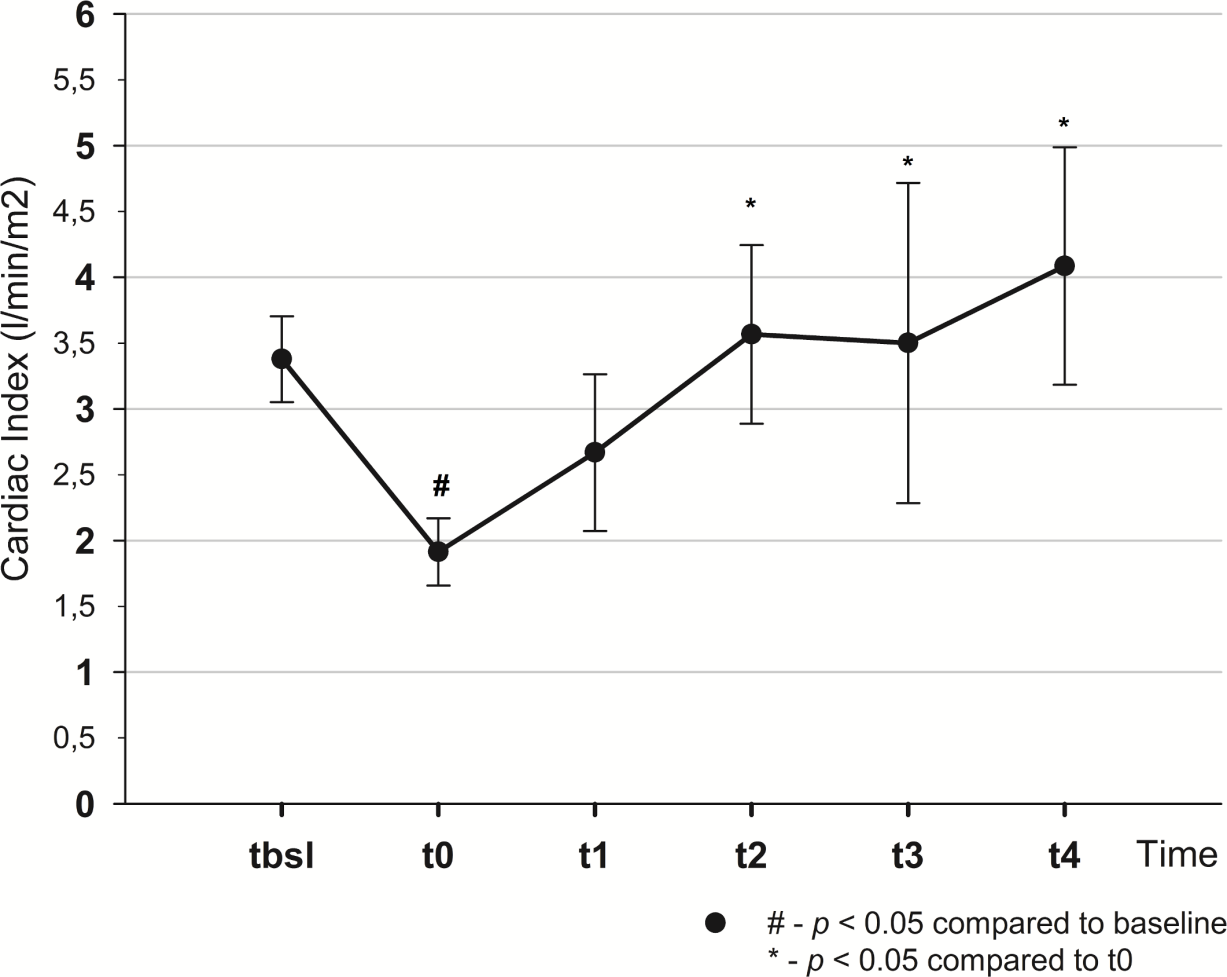
Changes of stroke volume index throughout the experiment.

**Fig 4:**
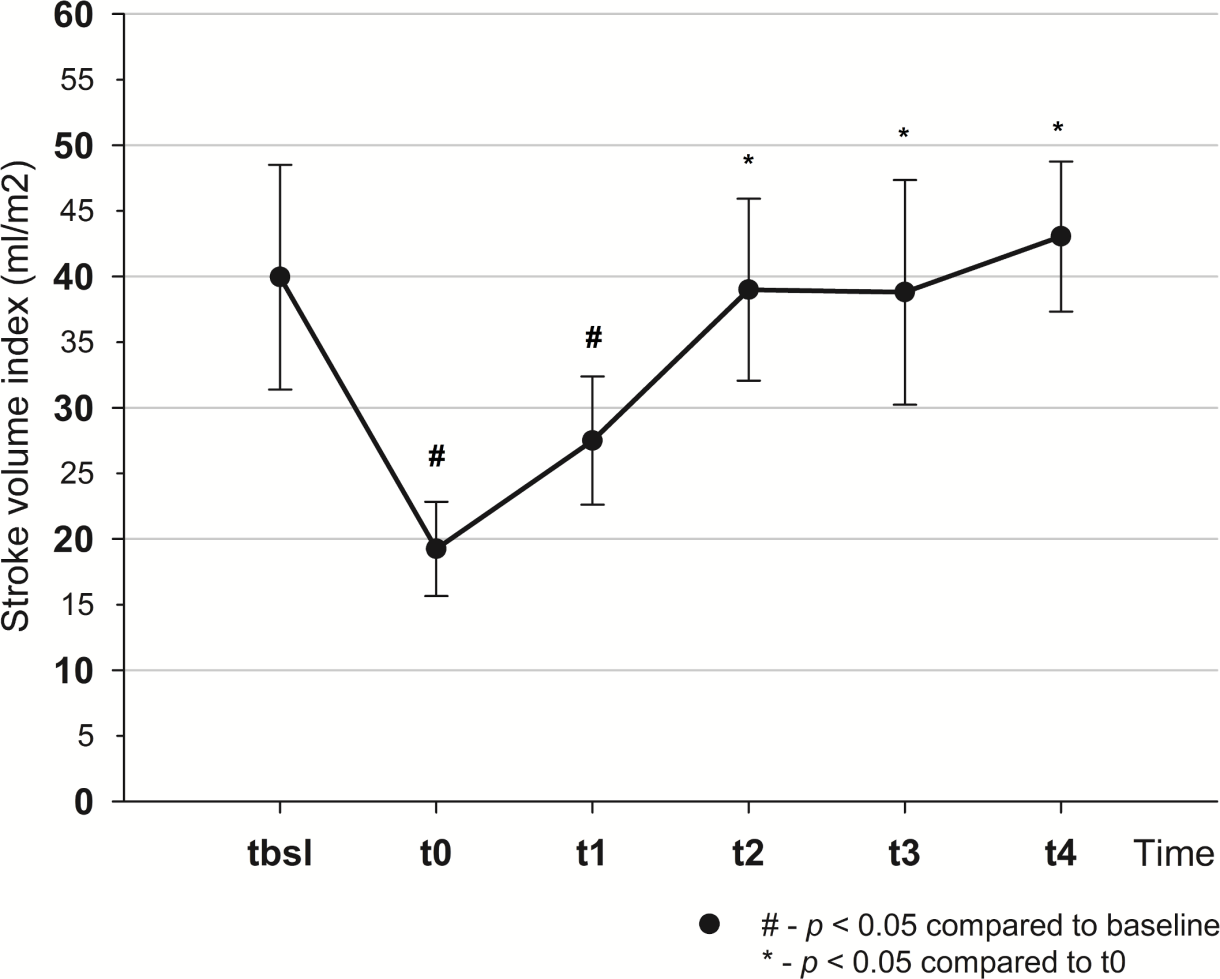
Changes of cardiac index throughout the experiment.

Cardiac index, SVI (Figs 3 and 4) and global end diastolic volume index (GEDVI) (Table 1) all decreased as a result of bleeding and returned to their initial values by t_2_.

Central venous pressure decreased during bleeding and increased during resuscitation but the changes did not reach statistical significance. There was a tendency towards a gradual increase in myocardial contractility as indicated by dPmax, which reached a significantly higher value by t_4_ compared to t_bsl_. Pulse contour analysis driven SVV and PPV increased from t_bsl_ to t_0_ and normalized by t_4_.

In general there was no significant correlation between MAP and the following hemodynamic parameters: MAP vs SVRI: r = 0.29.; SVRI vs HR: r = -0.22; SVRI vs SVI: r = -0.3; HR vs SVI: r = -0.67; MAP vs CI: r = 0.42; MAP vs SVI: r = 0.57; MAP vs VO_2_/DO_2_: r = 0.05.

### Changes in VO_2_ and DO_2_

The parameters of oxygen delivery and consumption are summarized in Table 1. Bleeding caused a decrease in DO_2_ and even though there was a steady improvement during resuscitation it remained lower throughout (t_2-4_) compared to t_bsl_. Hemoglobin levels showed a constant decline from t_bsl_ to the end of the experiment. Oxygen consumption increased after bleeding and almost doubled during the early phase of resuscitation. It remained elevated until the end of the experiment, however, it did not reach statistical significance as compared to baseline. Oxygen extraction ratio (as indicated by VO_2_/DO_2_) also increased during bleeding, and improved during resuscitation, but it remained significantly higher at t_4_ than at baseline. Lactate levels increased after bleeding (t_0_), then gradually decreased reaching baseline values by t_4_. ScvO_2_ decreased after bleeding. There was only a slight improvement by t_4_, and it remained significantly lower compared to t_bsl_.

## Discussion

The main findings of the current animal experiment on MAP-normalization-targeted resuscitation are the following. 1) Bleeding that caused 50% drop in SVI led to severe disturbances in DO_2_, VO_2_/DO_2_, ScvO_2_, CO_2_-gap and serum lactate levels. Although, this degree of bleeding also caused a significant drop in MAP, but it was less severe than changes in other hemodynamic indices and stabilized around 70–75 mmHg, which is still considered as adequate perfusion pressure. 2) Resuscitation resulted in the restoration of SVI and CI well before MAP normalized, hence further fluid administration seemed unnecessary. In fact almost 5 times more fluid was required as compared to the drained blood, suggesting gross fluid overloading whilst baseline MAP was still not reached. Therefore, our results provide further evidence that MAP-normalization is an inadequate end point to target fluid resuscitation during hemorrhage.

### MAP guided fluid resuscitation

With the invention of strain gauge pressure transducers and the simplified methods of radial arterial cannulation, continuous arterial pressure monitoring became available in the early 1960’s, and by 1990 more than 8 million arterial catheters have been placed [13]. Since that time generations of anesthesiologists, intensivists and critical care physicians have enjoyed the reassurance provided by the instantaneous blood pressure readings.

The confidence in numbers however implies the assumption that the perfusion pressure represents tissue perfusion, and this is far from the truth. Firstly, MAP is not a “universal perfusion pressure” for the various organs and tissues. Although perfusion is dependent on MAP, the distribution of blood flow is determined by regional arterial resistances, influenced by local metabolism, myogenic activity and flow-mediated vasodilation [14]. For example, in traumatic brain injury, the autoregulation of regional circulation deteriorates, rendering the conventional arterial pressure limits useless. Furthermore, in an otherwise healthy patient severe acute volume loss could be overlooked because blood pressure looks normal due to the compensatory sympathetic activity.

The term “normal blood pressure” is also difficult to define. Bijker et al. demonstrated that as many as 140 different hypotension definitions could be deciphered at the time of their study from papers published in leading anesthesiology journals. Due to this diversity, the incidence of hypotension in a real life cohort would vary between 5- and 99% [15].

Another approach could be based on observing the deviations from the patients’ usual values. However, in real-life emergency scenarios baseline values are often unavailable. In circumstances, where we do have initial blood pressure readings, for example at the beginning of surgery, these values can serve as baseline parameters to be followed as goals of treatment. However, our study provides important and convincing data that even a quasi-physiological baseline should not be regarded as the goal of resuscitation, or at least, one should be aware that a MAP-guided approach cannot guarantee the restoration of hemodynamic variables into their physiological ranges. Furthermore, our results also showed that after bleeding MAP still remained in a “normal” range (70–75 mmHg), and well above the recommended values of intervention (ie.: MAP~60–65 mmHg) by most guidelines (4). Our current findings are in accord with that of published earlier in similar animal experiments, when bleeding caused almost identical changes in blood pressure, t_bsl_:112±23 to t_0_: 74±18 mmHg, *p*<0.05 [16, 17]. Therefore, MAP may not only be an inappropriate resuscitation target but may also be an inadequate parameter to commence fluid resuscitation.

There is growing evidence that MAP is an inappropriate parameter to detect changes in CO and SV during fluid resuscitation [9, 18]. There are convincing data that MAP can remain stable over a wide range of changes of cardiac output and stroke volume [4, 7]. Our study also showed that during resuscitation stroke volume index and cardiac index together with other important indices, such as PPV, SVV, had already returned to their baseline values by t_2_ while MAP was still lower than the target. Therefore, fluid therapy had to be continued and more fluid was required to normalize MAP during the second part of the experiment (t_2_-t_4_), than initially (t_0_-t_2_): 1641.5 ± 799 ml vs 1080 ± 661 ml, *p* = 0.073. These results indicate that resuscitation could have been finished much earlier (at t_2_), when SV normalized, but had to be continued to reach baseline MAP, resulting in unnecessary fluid administration.

### Effects on VO_2_/DO_2_

The aim of resuscitation is to maintain adequate oxygen supply to the tissues. In our experiment, as predicted, DO_2_ decreased and VO_2_/DO_2_ increased after bleeding and showed improvement during fluid resuscitation. These changes were reflected in simple bedside tests such as ScvO_2_, CO_2_-gap and lactate levels, which are in accord with previously reported data [19, 20, 21, 22]. It is important to note, that although lactate levels normalized towards the end of the experiment—which can be due to either the resolution of anaerobic metabolism, or in part due to dilution of lactate by the large volume of infusion [23] -, but low ScvO_2_ still indicates some kind of imbalance between VO_2_/DO_2_, a phenomenon often seen at the bedside. This can be explained in part by the observation that VO_2_ remained significantly higher than at baseline, which may be due to the sympathetic response for bleeding. Regarding the relationship between MAP and VO_2_/DO_2_, it has been shown by several studies that correlation between these parameters is poor, which are in accord with our findings [12, 13, 24, 25].

### Which parameter should we follow during of fluid resuscitation?

Cardiac output is a major determinant of DO_2_, and several studies encourage optimization of cardiac output during fluid therapy [26, 27]. However, our previous series of animal experiments revealed that CO also has pitfalls as a therapeutic end-point [16, 17]. Comparing CO-, vs. SV-guided resuscitation, the latter provided sufficient restoration of bleeding caused hemodynamic disturbances, while CO normalized because of persistent tachycardia, resulting in residual hypovolemia [17].

However, we do not suggest that one single parameter is followed as the only guide for hemodynamic stabilization, but that a so called “multimodal, individualized concept” should be used, in which detailed evaluation of hemodynamic indices are put in the context of oxygen consumption, including ScvO_2_, venous-to-arterial pCO_2_-gap, lactate, etc. [28]. This approach provides the frame that enables us to fine tune treatment for the patient’s individual needs by putting them in the context of the complex puzzle that physiology is.

### Limitations

Despite the precisely defined circumstances of our protocol, our experiment has several limitations. First of all, a prospective randomized design (SVI-guided versus MAP-guided approach) would have provided more convincing results, and second, the results can only partially be extrapolated for real clinical settings. Reducing the SVI by 50% was a strictly controlled scenario, rarely happening in the everyday practice. Furthermore, animals may behave differently as compared to humans. This could explain that despite the blood loss, animals did not become severely hypotensive, neither tachycardic. It has previously been reported that hormonally active females tolerate hemorrhagic shock better as compared to males [29–31]. As 9 animals out of the 10 subjects were females it may have affected our results. Another limitation is that the rate of fluid administration was faster than it would have been in clinical practice, since baseline MAP was unable to be reached. Finally, it would have been useful to observe longer term effects of the applied fluid resuscitation strategy, but unfortunately it is not feasible in our animal laboratory at present.

## Conclusion

Mean arterial pressure is still considered the most frequently followed hemodynamic parameter during fluid resuscitation both in the operating theatre and on the ICU. Our results provide experimental evidence that pursuing a narrow range of mean arterial pressure as a single target gives false information about the status of fluid resuscitation as far as the complex hemodynamic picture is concerned, and may also cause harm such as fluid overload. On the ICU, the readily available complex multimodal monitoring facility enables the physicians to apply an individualized approach. This concept should replace the current practice of treating one or two single parameters only.
